# Supplementary material for: Harnessing many-body spin environment for long coherence storage and high-fidelity single-shot qubit readout
Source: Nat Commun. 2022 Jul 13;13:4048. doi: 10.1038/s41467-022-31618-4 (PMC9279416; doi:10.1038/s41467-022-31618-4)
Supplement: Supplementary file 1 — Supplementary Information [file 41467_2022_31618_MOESM1_ESM.pdf]

Supplementary Information: Harnessing many-body spin environment for long coherence storage and high-fidelity single-shot qubit readout

George Gillard, Edmund Clarke, and Evgeny A. Chekhovich

Supplementary Note 1. ELECTRON-NUCLEAR SPIN SYSTEM OF A QUANTUM DOT.

The Hamiltonian of the electron central spin $\mathbf{s}$ and the nuclear spin bath $\mathbf{I}_j$, where $1 \leq j \leq N$ and N is the number of nuclei in the dot, can be written as the following sum of terms:

$$\mathcal{H} = \mathcal{H}_{\mathrm{Z,N}} + \mathcal{H}_{\mathrm{Q,N}} + \mathcal{H}_{\mathrm{DD,N}} + \mathcal{H}_{\mathrm{Z,e}} + \mathcal{H}_{\mathrm{hf}} + \mathcal{H}_{\mathrm{Env,e}} \quad (1)$$

The first Zeeman term describes interaction of the QD nuclear spins $\mathbf{I}_j$ with the static magnetic field B_{z} aligned along the z axis:

$$\mathcal{H}_{\mathrm{Z,N}} = -\sum_j \hbar\gamma_j B_{\mathrm{z}} \hat{I}_{\mathrm{z},j}, \quad (2)$$

where $\hbar = h/(2\pi)$ is the reduced Planck constant, γ_j is the gyromagnetic ratio of the j-th nuclear spin and $\hat{\mathbf{I}}_j$ is a vector of spin operators with Cartesian components $(\hat{I}_{\mathrm{x},j}, \hat{I}_{\mathrm{y},j}, \hat{I}_{\mathrm{z},j})$. The result of the Zeeman term alone is a spectrum of equidistant single-spin energies $\hbar\gamma_j B_{\mathrm{z}} I_{\mathrm{z}}$, corresponding to $2I+1$ eigenstates with nuclear spin z-projections I_{z}, where $-I \leq I_{\mathrm{z}} \leq +I$. The interaction of the nuclear electric quadrupolar moment with the electric field gradients is described by the term (Ch. 10 in Ref. [1]):

$$\mathcal{H}_{\mathrm{Q,N}} = \sum_j \frac{q_j}{6}[3\hat{I}^2_{\mathrm{z}',j} - I_j^2 + \eta_j(\hat{I}^2_{\mathrm{x}',j} - \hat{I}^2_{\mathrm{y}',j})], \quad (3)$$

where q_j and η_j describe the magnitude and asymmetry of the electric field gradient tensor, whose principal axes are $x'y'z'$. In self-assembled QDs the electric field gradients at the nuclear sites are as large as $q_j/h \approx 10$ MHz and are dominated by the elastic strains [2, 3]. The strain is inhomogeneous within the QD volume, so that q_j and η_j vary strongly between individual nuclei. The axes $x'y'z'$ are different for each nucleus and generally do not coincide with crystalographic axes or magnetic field direction. At sufficiently strong magnetic fields $|\hbar\gamma_j B_{\mathrm{z}}| \gg |q_j|$, quadrupolar effects can be treated perturbatively – the main effect is the nonharmonicity of the nuclear spin energies and the resulting splitting of the NMR spectrum into a quadrupolar multiplet of $2I$ distinct transitions. The $I_{\mathrm{z}} = \pm 1/2$ states of a half-integer nuclear spin are influenced by quadrupolar effects only in the second order, resulting in a smaller inhomogeneous broadening, compared to the broadening of

the $|I_\mathrm{z}| > 1/2$ state energies. This allows for spectral isolation of the $I_\mathrm{z} = \pm 1/2$ subspaces, which can then be treated as effective spin-1/2 nuclei. Within the $I_\mathrm{z} = \pm 1/2$ subspace, the quadrupolar Hamiltonian is identical to the Zeeman term, so that we can replace $\mathcal{H}_\mathrm{Q,N} \rightarrow -\sum_j \hbar\gamma_j \Delta B_\mathrm{z} \hat{I}_{\mathrm{z},j}$, where inhomogeneous quadrupolar shifts q_j are emulated by magnetic field inhomogeneity on the scale of $\Delta B_\mathrm{z,j} \propto q_j^2/(\hbar^2\gamma_j^2 B_\mathrm{z})$.

Direct interaction between the nuclei is described by the dipole-dipole Hamiltonian:

$$\mathcal{H}_\mathrm{DD} = \sum_{j<k} b_{j,k} \left(3\hat{I}_{\mathrm{z},j}\hat{I}_{\mathrm{z},k} - \hat{\mathbf{I}}_j \cdot \hat{\mathbf{I}}_k\right),$$
$$b_{j,k} = \frac{\mu_0}{4\pi}\frac{\gamma_j\gamma_k}{2}\frac{1 - 3\cos^2\theta_{j,k}}{r_{j,k}^3} \tag{4}$$

Here, $\mu_0 = 4\pi \times 10^{-7}$ NA^{-2} is the magnetic constant and $r_{j,k}$ denotes the length of the vector, which forms an angle θ with the z axis and connects the two spins j and k. The typical magnitude of the interaction constants for the nearby nuclei in InGaAs is $\max(|b_{j,k}|)/h \approx 100$ Hz. The Hamiltonian of Supplementary Eq. (4) has been truncated to eliminate all spin non-conserving terms – this is justified for static magnetic field exceeding $\gtrsim 1$ mT. Due to its bilinear form in terms of spin operators, the dipolar interaction is not refocused by the nuclear spin echo sequences, and is responsible for collective nuclear spin decoherence on a timescale $T_\mathrm{2,N,DD} \propto h/\max(|b_{j,k}|)$.

The electron Zeeman term is

$$\mathcal{H}_\mathrm{Z,e} = \mu_\mathrm{B} g_\mathrm{e} B_\mathrm{z} \hat{s}_\mathrm{z}, \tag{5}$$

where μ_B is the Bohr magneton and g_e is the effective conduction-band electron g-factor, estimated to be $g_\mathrm{e} \approx -0.63$ in the studied QDs.

The interaction of the conduction band electron spin $\mathbf{s}$ with the ensemble of the QD nuclear spins is dominated by the contact (Fermi) hyperfine interaction, with the following Hamiltonian:

$$\mathcal{H}_\mathrm{hf} = \sum_j A_j(\hat{s}_\mathrm{x}\hat{I}_{\mathrm{x},j} + \hat{s}_\mathrm{y}\hat{I}_{\mathrm{y},j} + \hat{s}_\mathrm{z}\hat{I}_{\mathrm{z},j}), \tag{6}$$

where the hyperfine constant of an individual nucleus j is $A_j = A^{(j)}|\psi(\mathbf{r}_j)|^2 v$. Unlike A_j, the $A^{(j)}$ hyperfine constant is a parameter dependent only on the material and the isotope type to which nucleus j belongs, $|\psi(\mathbf{r}_j)|^2$ is the density of the electron envelope wavefunction at the nuclear site $\mathbf{r}_j$ of the zinc blende crystal lattice, and v is the crystal volume per one cation or one anion. The definitions of the hyperfine constants differ between different sources. With the definition adopted here, a fully polarized isotope with spin I, hyperfine constant A and a 100% abundance (e.g. ^{75}As),

would shift the energies of the electron spin states $s_z = \pm 1/2$ by $\pm AI/2$, irrespective of the shape of $|\psi(\mathbf{r}_j)|^2$. In that case, the typical values in InGaAs are $A \approx 50$ μeV (Refs. [4–6]).

The three orders of magnitude disparity in the energy scales of $\mathcal{H}_{\mathrm{Z,e}}$ and $\mathcal{H}_{\mathrm{Z,N}}$ suppresses at high magnetic field the direct (first-order) electron nuclear spin flip-flops governed by the $\propto \hat{s}_x \hat{I}_{x,j} + \hat{s}_y \hat{I}_{y,j}$ term. The remaining secular term $\hat{s}_z \hat{I}_{z,j}$ is responsible for the Knight shifts of the nuclear magnetic resonance frequencies and the electron spin hyperfine shift (Overhauser shift) E_{hf}. In the second order perturbation expansion, $\mathcal{H}_{\mathrm{hf}}$ gives rise to hyperfine-mediated nuclear-nuclear spin flip-flops. The effective coupling strength of the two nuclei j and k is $\propto A_j A_k / \Delta E_{\mathrm{Z}}$, where ΔE_{Z} is the energy splitting of the electron spin levels, which includes both the Zeeman splitting $\mu_{\mathrm{B}} g_{\mathrm{e}} B_{\mathrm{z}}$ and the electron hyperfine shifts E_{hf} due to the polarized nuclei [7]. Thus, unlike the Knight shifts, the hyperfine-mediated nuclear-nuclear interaction is field dependent, and is expected to have the most pronounced effect, such as shorter nuclear spin coherence, at low magnetic fields [8].

The coupling of the electron spin to external environments is described by the term $\mathcal{H}_{\mathrm{Env,e}}$ in the total Hamiltonian of Supplementary Eq. (1). Interaction of the electron spin with phonons, mediated by spin-orbit coupling, induces electron spin relaxation with rate $\mathit{\Gamma}_{\mathrm{e}} \propto B_{\mathrm{z}}^4$ (or $\mathit{\Gamma}_{\mathrm{e}} \propto B_{\mathrm{z}}^5$ at low temperatures), strongly dependent on magnetic field B_{z} (Ref. [9]). Cotunneling coupling between the quantum dot and the nearby electron Fermi reservoir also gives rise to QD electron spin relaxation, whose rate depends weakly on magnetic field, but is strongly affected by the gate bias V_{G} that controls the energies of the QD states with respect to the Fermi level [9]. Additional electron spin relaxation mechanisms may arise for example from the charge fluctuations of the defects in proximity to the QD. Full description of electron spin dynamics is a complex problem. However, as we show in this work, in order to explain the collective coherence of the QD nuclear spin bath, it is sufficient to use a simplified spectral diffusion model, where environment-driven electron spin flips are treated as random events, described by the experimentally measurable electron spin relaxation rate $\mathit{\Gamma}_{\mathrm{e}}$.

Supplementary Note 2. SPECTRAL DIFFUSION MODEL.

We consider evolution of the nuclear spin ensemble in the frame rotating at nuclear Larmor frequency determined by the strong static magnetic field B_{z}. Since all NMR experiments are conducted on the $(I_{\mathrm{z}} = -1/2) \leftrightarrow (I_{\mathrm{z}} = +1/2)$ transitions, the nuclei can be treated for simplicity as spin-1/2 particles. Optical cooling is used to create nonequilibrium longitudinal nuclear spin magnetization along the z axis. The initial $(\pi/2)_{\mathrm{x}}$ rf pulse of the sequence flips the spins, so

that magnetization points along the equatorial x axis of the rotating frame. In the absence of any interactions, the spins remain static in the rotating frame indefinitely, corresponding to unperturbed Larmor precession in the laboratory frame. Various interactions cause dephasing and decoherence of the nuclear spin ensemble – here we focus on the effect of the central electron spin $\mathbf{s}$ coupled to the nuclear spins $\mathbf{I}_j$ via contact hyperfine interaction (see Supplementary Note 1). With large static magnetic field applied, electron-nuclear flip-flops are energetically forbidden, truncating the hyperfine Hamiltonian to $A_j \hat{s}_\mathrm{z} \hat{I}_{\mathrm{z},j}$. Here A_j is the hyperfine constant of the j-th nucleus, which is proportional to the electron envelope wavefunction density $A_j \propto |\psi(\mathbf{r}_\mathrm{j})|^\mathbf{2}$ at the site $\mathbf{r}_\mathrm{j}$ of the nucleus. We assume that the electron can only occupy two discrete spin states with z-projections $s_\mathrm{z} = +1/2$ ($\uparrow$) or $s_\mathrm{z} = -1/2$ ($\downarrow$). For each nucleus j, the hyperfine field of the electron gives rise to a Knight shift $\nu_{\mathrm{e},j} = A_j s_\mathrm{z}/h$ of the NMR frequency. For a static electron spin and in the absence of nuclear-nuclear interactions, the π pulse of the nuclear spin echo sequence $(\pi/2)_\mathrm{x} - (\tau_\mathrm{evol}/2) - (\pi)_\mathrm{x} - (\tau_\mathrm{evol}/2) - (\pi/2)_\mathrm{x}$ can completely refocus the effect of inhomogeneous ($A_j \neq A_k$) Knight shifts resulting in an echo in transverse nuclear spin magnetization at time τ_evol after the initial $\pi/2$ pulse. It is the temporal evolution of the electron spin $s_\mathrm{z}(t)$ during the nuclear spin evolution that leads to irreversible decay of the echo amplitude through decoherence.

In order to model the time evolution of the electron spin we assume that it is governed by a memoryless random telegraph process. We denote the rate of transition from $\uparrow$ to $\downarrow$ as $w_{\uparrow\rightarrow\downarrow} = \mathit{\Gamma}_\mathrm{e}(1 - \rho_\mathrm{e})/2$ and the rate of the reverse process as $w_{\downarrow\rightarrow\uparrow} = \mathit{\Gamma}_\mathrm{e}(1 + \rho_\mathrm{e})/2$. The stationary population probabilities are then $p_\uparrow = (1 + \rho_\mathrm{e})/2$ and $p_\downarrow = (1 - \rho_\mathrm{e})/2$. Here we express the quantities in terms of the experimentally measurable electron spin relaxation rate $\mathit{\Gamma}_\mathrm{e}$ ($\mathit{\Gamma}_\mathrm{e} = 1/T_{1,\mathrm{e}}$, where $T_{1,\mathrm{e}}$ is the electron spin lifetime) and the equilibrium polarization degree $\rho_\mathrm{e} = \tanh[-\mu_\mathrm{B} g_\mathrm{e} B_\mathrm{z}/(2k_\mathrm{B}T)]$ of an electron spin with g-factor g_e at a temperature T (k_B is the Boltzmann constant). Due to the electron spin flips the Knight shift experienced by each nucleus is a random process $\nu_{\mathrm{e},j}(t) = A_j s_\mathrm{z}(t)/h$. In order to evaluate the relative spin echo amplitude of an individual nuclear spin we consider the total rotation of the magnetization vector in the xy plane of the rotating frame caused by the Knight shift, and find $\cos[2\pi \int_0^{\tau_\mathrm{evol}/2} \nu_{\mathrm{e},j}(t) dt - 2\pi \int_{\tau_\mathrm{evol}/2}^{\tau_\mathrm{evol}} \nu_{\mathrm{e},j}(t) dt]$. The echo amplitude observed in optically detected NMR is a sum of contributions from the individual nuclei, weighted by the electron wavefunction density. Moreover, we take the average over all possible implementations of the random process $s_\mathrm{z}(t)$. The amplitude of the QD nuclear spin

echo as a function of free evolution time $\tau_{\rm evol}$ is then modeled as

$$\mathcal{E}(\tau_{\rm evol}) = \left(N_{\rm hist}\sum_{j=1}^{N} A_j\right)^{-1} \sum_{i=1}^{N_{\rm hist}}\sum_{j=1}^{N} A_j \cos[\frac{2\pi A_j}{h}\left(\int_0^{\frac{\tau_{\rm evol}}{2}} s_{{\rm z},i}(t)dt - \int_{\frac{\tau_{\rm evol}}{2}}^{\tau_{\rm evol}} s_{{\rm z},i}(t)dt\right)], \quad (7)$$

The integral over $t = [\tau_{\rm evol}/2, \tau_{\rm evol}]$ is taken with a negative sign since the reversal of the nuclear spin magnetization by the π pulse at $t = \tau_{\rm evol}/2$ is equivalent to the change in the direction of the nuclear spin precession. The contributions of the N QD nuclei are weighted by $A_j \propto |\psi(\mathbf{r}_{\rm j})|^{\mathbf{2}}$ to describe the optical detection of NMR via electron hyperfine shifts $E_{\rm hf}$. The sum over i implements the averaging over a finite number $N_{\rm hist}$ of electron spin evolution histories $s_{{\rm z},i}(t)$. The ideal echo amplitude at zero free evolution time is $\mathcal{E}(0) = 1$.

There is no universal closed-form solution to Supplementary Eq. (7). A simple approximation of $E(\tau_{\rm evol})$ can be found in the limiting case, relevant to experimental results of this work. If the rate of electron spin flip events $= \mathit{\Gamma}_{\rm e}/2$ is small compared to a typical Knight shift $\nu_{\rm e}$, then one or more flips of the electron spin are likely to lead to a complete loss of the nuclear spin echo. Indeed, unless the electron spin flip occurs within a short time interval $\Delta t \approx T_{\rm 2,N}^{*,(1e)} \propto 1/(2\pi\delta\nu_{\rm e})$ near the start or the end of the nuclear spin echo sequences, the precession phases acquired by the nuclear spins before and after the electron spin flip do not cancel out, and no echo is observed. The same reasoning holds for multiple electron spin flips during the nuclear spin echo sequence. Thus the formation of the collective nuclear spin echo is conditional on no electron spin flips occurring during the entire nuclear spin evolution. In equilibrium, the probability of having no electron spin flips in a short time period dt is $1 - w_{\uparrow\rightarrow\downarrow}p_{\uparrow}dt - w_{\downarrow\rightarrow\uparrow}p_{\downarrow}dt$. For a finite evolution time $\tau_{\rm evol}$ the probability to have no electron spin flips is then $\lim_{dt\rightarrow 0}(1 - w_{\uparrow\rightarrow\downarrow}p_{\uparrow}dt - w_{\downarrow\rightarrow\uparrow}p_{\downarrow}dt)^{\tau_{\rm evol}/dt} = \exp[-(w_{\uparrow\rightarrow\downarrow}p_{\uparrow} + w_{\downarrow\rightarrow\uparrow}p_{\downarrow})\tau_{\rm evol}] = \exp(-\mathit{\Gamma}_{\rm e}(1-\rho_{\rm e}^2)\tau_{\rm evol}/2)$. For this regime of spectral diffusion under slow electron central spin flips ($\mathit{\Gamma}_{\rm e}/\delta\nu_{\rm e} \ll 1$), averaging over electron spin-flip histories leads to an exponential decay of the echo amplitude with a characteristic coherence time $T_{\rm 2,N,SD} \propto 2/(\mathit{\Gamma}_{\rm e}(1-\rho_{\rm e}^2)) = 2T_{\rm 1,e}/(1-\rho_{\rm e}^2)$, linearly proportional to the central spin lifetime $T_{\rm 1,e}$.

We perform numerical evaluation of the full Supplementary Equation (7) to compute $T_{\rm 2,N,SD}$ for arbitrary $\mathit{\Gamma}_{\rm e}$, $\rho_{\rm e}$ and $\nu_{{\rm e},j}$ without any simplifying assumptions about $\mathit{\Gamma}_{\rm e}$. We use $N_{\rm hist} \geq 10000$ and the set of the Knight shifts $\nu_{{\rm e},j}$ for nuclei within the quantum dot is modeled by drawing $N = 1000$ samples from a random distribution, and then taking the absolute values to ensure all $\nu_{{\rm e},j}$ are positive. We use uniform or Gaussian distribution – for the practically relevant slow-electron-flip regime the root mean square broadening $\delta\nu_{\rm e} = \sqrt{\langle\nu_{{\rm e},j}^2\rangle}$ and the actual shape of the distribution make no difference (here, $\langle\rangle$ denotes averaging over QD nuclei). The dependence of $T_{\rm 2,N,SD}$ on $\mathit{\Gamma}_{\rm e}/\delta\nu_{\rm e}$ is shown in Supplementary Fig. 1 for the case of uniform distribution. In the

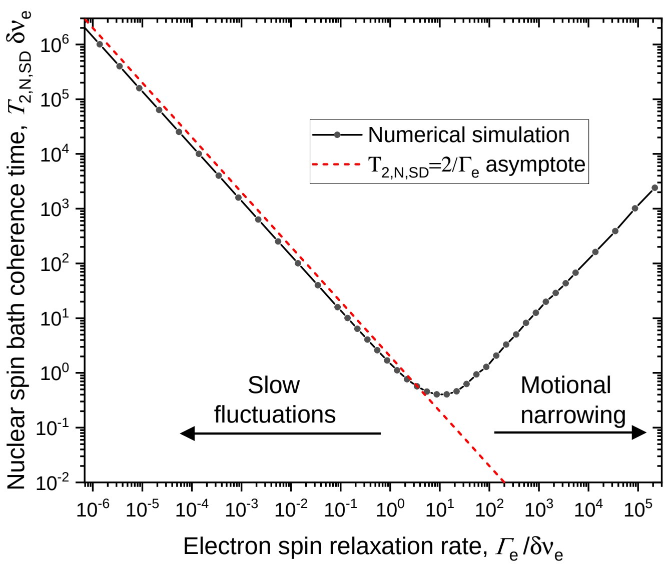

Supplementary Figure 1. **Numerical simulation of decoherence induced by spectral diffusion.** Coherence time $T_{2,\mathrm{N,SD}}$ of the nuclear spin bath as a function of the relaxation rate $\mathit{\Gamma}_\mathrm{e} = 1/T_{1,\mathrm{e}}$ of the central electron spin. Results are for an unpolarized electron $\rho_\mathrm{e} = 0$. The characteristic root mean square width $\delta\nu_\mathrm{e}$ of the uniform distribution of the Knight shifts is used to scale $T_{2,\mathrm{N,SD}}$ and $\mathit{\Gamma}_\mathrm{e}$ and plot dimensionless values on both axes.

slow-electron-flip limit ($\mathit{\Gamma}_\mathrm{e}/\delta\nu_\mathrm{e} \ll 1$), we find $T_{2,\mathrm{N,SD}} \approx 1.38T_{1,\mathrm{e}}/(1-\rho_\mathrm{e}^2)$, in good agreement with a simple estimate presented in the paragraph above. At $\mathit{\Gamma}_\mathrm{e}/\delta\nu_\mathrm{e} \approx 10$, the spin bath decoherence time reaches its minimum $T_{2,\mathrm{N,SD}} \approx 0.4/\delta\nu_\mathrm{e}$. Further increase of the electron spin flip rate $\mathit{\Gamma}_\mathrm{e}/\delta\nu_\mathrm{e} \gg 1$ results in an increasing $T_{2,\mathrm{N,SD}}$: In this motional narrowing regime the electron flips take place faster than it takes the nuclei to make any significant precession under the effect of the Knight shift, so that the Knight shifts are effectively averaged to zero, leading to an extended spin bath coherence time $T_{2,\mathrm{N,SD}}$.

Dipole-dipole coupling between the nuclei of an empty QD ($0e$) itself causes spin bath decoherence [10–12] with a characteristic time $T_{2,\mathrm{N,DD}} \propto h/\max(|b_{j,k}|)$. In order to model the experimentally measured nuclear spin coherence time $T_{2,\mathrm{N}}^{(1e)}$ in presence of an electron we combine the dipole-dipole and spectral diffusion decoherence rates $1/T_{2,\mathrm{N}}^{(1e)} = 1/T_{2,\mathrm{N,DD}} + 1/T_{2,\mathrm{N,SD}}$. The dipolar decoherence time $T_{2,\mathrm{N,DD}}$ is taken to be the measured empty-QD ($0e$) time $T_{2,\mathrm{N}}^{(0e)}$, which is nearly independent of magnetic field in the studied structures. We can then write the model equation for $T_{2,\mathrm{N}}^{(1e)}$ in a closed form:

$$1/T_{2,\mathrm{N}}^{(1e)} = 1/T_{2,\mathrm{N}}^{(0e)} + (1 - \tanh^2[-\frac{\mu_\mathrm{B} g_\mathrm{e} B_\mathrm{z}}{2k_\mathrm{B}T}])/(1.38T_{1,\mathrm{e}}), \tag{8}$$

where all quantities are either measurable ($g_{\rm e}$, $T_{1,\rm e}(B_{\rm z})$, $T_{2,\rm N}^{(0e)}$) or controlled in the experiment ($B_{\rm z}$, T). Equation (1) of the main text is a special case of this Supplementary Eq. (8) in the high-temperature or low-field limit where equilibrium electron spin polarization $\rho_{\rm e}$ is small. The model plots of Fig. 2b in the main text employ Supplementary Eq. (8) and therefore take into account the electron spin polarization, although the difference with the simplified model of $\rho_{\rm e} \approx 0$ is relatively small for the studied range of magnetic fields $B_{\rm z} < 8$ T at $T \approx 4.5$ K. We also perform a simple fit using an empirical power-law model $1/T_{2,\rm N}^{(1e)} = 1/T_{2,\rm N}^{(0e)} + 1/(a_{\rm SD} B_{\rm z}^{k_{\rm SD}})$. The best-fit exponent $k_{\rm SD} = -3.67 \pm 2.33$ (90% confidence level) agrees with the -4 exponent expected for the phonon-related mechanism at high field. The accuracy of $k_{\rm SD}$ is limited by the range of magnetic fields $B_{\rm z} \leq 8$ T available in this work.

Supplementary Note 3. DETAILS OF PULSED NMR IMPLEMENTATION

A schematic of the timing sequence used in the pulsed NMR experiments is shown in Supplementary Fig. 2. The gate bias $V_{\rm G}$ applied to the structure is varied together with optical and radio frequency pulses in order to provide optimal charging conditions for each stage. High power ($P_{\rm Pump} \approx 8$ mW) circularly polarized optical excitation with a wavelength $\lambda \approx 850$ nm, corresponding to the ground states of the InAs wetting layer, is applied for pumping time $T_{\rm Pump} \approx 8$ s. Such optical pumping produces a state of the QD nuclear spin ensemble that is well described by a Boltzmann distribution [6]. A bias of $V_{\rm Pump} = -1.5$ V to 1.0 V applied during optical pumping produces large dynamic nuclear polarisation (DNP), characterised by the hyperfine shifts of up to $E_{\rm hf} \approx \pm 120$ μeV. It is found that negative values of $V_{\rm Pump}$ generate a somewhat reduced DNP but produce the most stable results, whereas $V_{\rm Pump}$ exceeding the single-electron Coulomb blockade bias V_{1e} leads to intermittent ejection of the electron or capture of an additional electron during the application of the high power rf pulses. This undesired effect is found to be particularly pronounced in the thickest barrier sample with $t_{\rm B} = 52$ nm. In order to avoid this parasitic charging we use $V_{\rm Pump} = -1.5$ V in all measurements, and the presence of the electron in a QD during the NMR sequences can be verified directly by observing a bimodal distribution in single-shot measurements, such as those shown in Fig. 3c of the main text. A short delay $T_{\rm Del} \approx 5$ ms is introduced between switching the gate bias and the optical excitation, in order to account for delays of the mechanical shutters implementing the pump and probe laser pulses.

Frequency swept rf excitation is applied after optical pumping to implement adiabatic population transfer [13], which transforms the $I_{\rm z} = \pm 3/2$ nuclear spin states into the $I_{\rm z} = \pm 1/2$

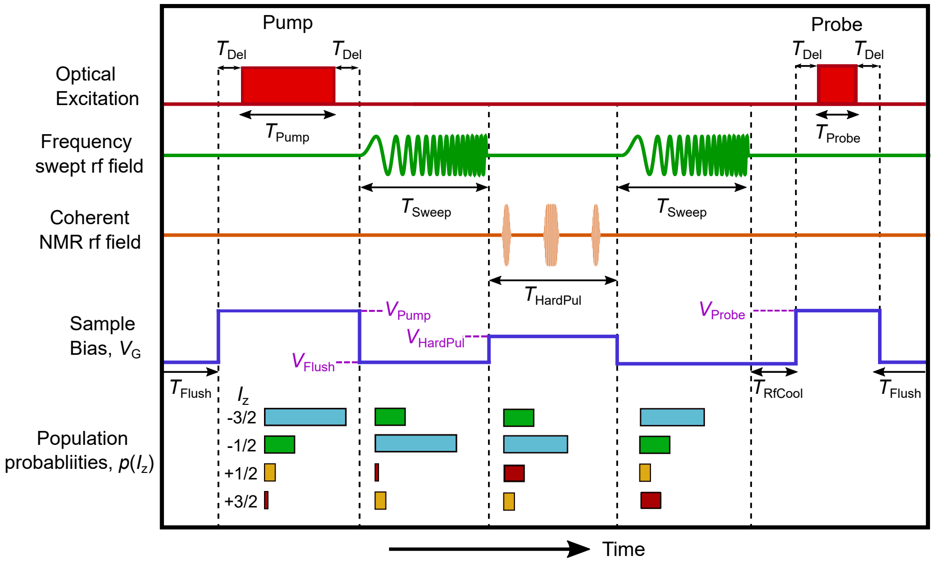

Supplementary Figure 2. **Pulsed NMR measurement timing diagram.** Timing sequence of one cycle used in pulsed NMR experiments on $I_z = \pm 1/2$ spin states of half-integer quadrupolar nuclei. Optical excitation includes pump and probe laser pulses (red), frequency swept rf implementing adiabatic population transfer (green), high power pulsed rf implementing coherent control of the nuclear spins (orange) and applied sample bias V_G controlling the charge state of the QD (blue). The bars in the bottom row sketch the population probabilities $p(I_z)$ of the spin states I_z at the end of each stage of the cycle. For discussion see Supplementary Note 3.

states. This increases the difference in the number of $I_z = -1/2$ and $I_z = +1/2$ nuclei that are coherently controlled under pulsed rf excitation that follows. The frequency sweeps start 5 to 10 MHz away from the $-1/2 \leftrightarrow +1/2$ central transition (CT) frequency and end 50 to 200 kHz near the CT frequency, depending on the isotope. These sweep ranges are chosen to fully cover the strain-broadened satellite transitions (STs) $-3/2 \leftrightarrow -1/2$ and $+1/2 \leftrightarrow +3/2$, while maintaining sufficient detuning from the CT frequency. A frequency sweep rate ranging from 5 to 10 MHz s^{-1} is used, depending on the isotope, to achieve adiabatic inversion. Adiabatic sweeps are applied while the sample is kept at large reverse bias $V_{\text{Flush}} = -1.5$ V to ensure any charges generated during optical pumping are flushed out of the QD leaving it empty ($0e$ state). High power radio frequency pulses are then applied while the sample is at an arbitrary bias V_{HardPul} to study nuclear spin bath coherence under various QD charge states and tunneling conditions. The same adiabatic sweeps that precede the high power rf sequence are applied in some measurements once again afterwards, transferring the final populations of the $I_z = \pm 1/2$ states back into $I_z = \pm 3/2$ states, respectively. This provides a factor of 3 increase in the optically detected hyperfine shift variations ΔE_{hf} at the

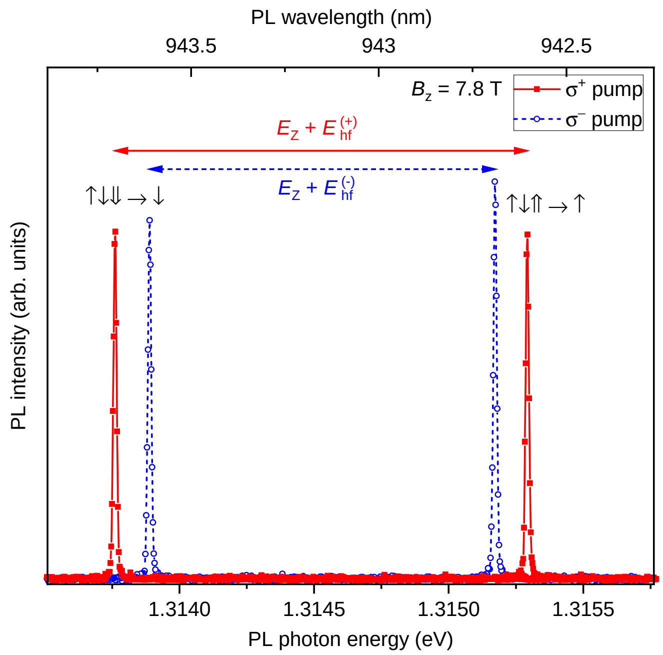

Supplementary Figure 3. **Hyperfine shifts in quantum dot photoluminescence spectra**. High resolution PL spectra of a negatively charged X^- trion following σ^+ (squares) or σ^- (circles) circularly polarized optical pumping, which creates electrons with opposite spin polarizations. The electrons transfer their spin to the nuclei via magnetic (hyperfine) interaction, resulting in a buildup of net nuclear spin polarization with opposite signs for σ^+ and σ^- pumping. Through the same hyperfine interaction, the average nuclear spin polarization shifts the energies of the $\downarrow$ and $\uparrow$ electrons left after recombination of a negatively charged trion with a hole with total angular momentum projection of $j_z = -3/2$ ($\Downarrow$) or $j_z = +3/2$ ($\Uparrow$), respectively.

expense of a longer experimental cycle. After all the rf excitations have completed, a short delay of $T_{\rm RfCool} \approx 60$ ms at $V_{\rm Flush}$ is used to ensure dissipation of any rf-induced heat before optical probing is done.

Optical probe is a low power ($P_{\rm Probe} = 1-10\ \mu$W) laser pulse of duration $T_{\rm Probe} = 5-20$ ms, at a bias of $V_{\rm Probe} \approx 0.3-0.65$ V, chosen to maximise optical photoluminescence (PL) intensity of the negatively charged trion X^-. After each cycle the sample is returned to the flush bias $V_{\rm Flush}$ for a time $T_{\rm Flush} = 30$ ms to remove any residual charges before the next cycle begins. Supplementary Fig. 3 shows typical probe PL spectra measured at different nuclear spin polarization states, induced by optical pumping with σ^+ and σ^- polarized light. Each spectrum is a doublet. The high energy peak of the doublet originates from recombination of a trion with a spin-up hole ($\Uparrow$), leaving a spin-up electron ($\uparrow$) in the dot. Conversely, the low energy peak is due to the trion with a spin-down hole ($\Downarrow$), leaving a spin-down electron ($\downarrow$). The splitting of the spectral doublet is the

sum of the nuclear-spin-independent Zeeman splitting E_Z and the nuclear-spin-related hyperfine shift E_{hf}. By subtracting the spectral splittings measured under two different nuclear spin states, E_Z is eliminated to obtain a hyperfine shift variation, which is $\Delta E_{hf} = E_{hf}^{(+)} - E_{hf}^{(-)}$ for the spectra shown in Supplementary Fig. 3. This differential technique is used in all pulsed magnetic resonance measurements. For example, in a spin echo experiment with averaging over multiple pump-rf-probe cycles, we conduct two measurements, different only in the choice of the final pulse $(\pi/2)_x$ or $(\pi/2)_{-x}$, which converts the same transverse nuclear spin polarization into negative or positive hyperfine splitting, respectively. By subtracting the results of the two measurements, we obtain pure spin echo amplitudes, such as shown in Fig. 1f of the main text. The typical error in ΔE_{hf} is less than 1 μeV, and is determined by the signal to noise ratio of the optical detection system. Since the hyperfine shift is a sum of the effects of $N \approx 4 \times 10^4$ nuclei, the statistical noise $\approx \Delta E_{hf}/\sqrt{N}$ is much smaller, on the order of 0.05 μeV for a typical $\Delta E_{hf} \approx 10$ μeV.

Supplementary Note 4. ADDITIONAL RESULTS FOR ^{75}As NUCLEAR SPINS

Supplementary Fig. 4 shows the results of bias dependent Rabi oscillation measurements. Clear oscillations with a period of ≈ 4.4 μs are observed. At $V_G \approx 0.4$ V, corresponding to single-electron charging of the QD, the damping of the Rabi oscillations is accelerated by the inhomogeneous Knight shifts.

Supplementary Fig. 5 shows the results of free induction decay NMR measured using the spin echo sequences with a fixed τ_0 and variable τ. The experiments are similar to those presented in Figs. 1d, e of the main text for ^{69}Ga. The time resolved measurements with a sequence $(\pi/2)_x - \tau_0 - (\pi)_x - \tau - (\pi/2)_x$ shown in Supplementary Fig. 5a provide estimates of the nuclear spin dephasing time $T_{2,N}^*$. In order to derive the spectral information we also implement the $(\pi/2)_x - \tau_0 - (\pi)_x - \tau - (\pi/2)_y$ sequence. The rotation by the $(\pi/2)_y$ pulse is around the axes orthogonal to the rotation axis of the $(\pi/2)_x$ pulse. Thus the sequences with the $(\pi/2)_y$ final pulse measures the quadrature component of the echo nuclear spin polarization. By performing the Fourier transform on both the in-phase and the quadrature data we obtain the NMR lineshapes such as shown in Supplementary Fig. 5b and Fig. 1e of the main text. The lineshapes are then fitted with compressed exponential profiles to derive the full width at half maximum (FWHM) spectral broadening.

For ^{69}Ga at $B_z = 7.8$ T we find $T_{2,N}^{*,(0e)} \approx 34.6$ μs in an empty QD, and a corresponding spectral profile with a FWHM of ≈ 13.3 kHz and a compression parameter $\eta \approx 1.6$ (Figs. 1e, d of the main text). For ^{75}As at $B_z = 7.8$ T (Supplementary Fig. 5) we find $T_{2,N}^{*,(0e)} \approx 7.4$ μs, $\eta \approx 1.38$, and a

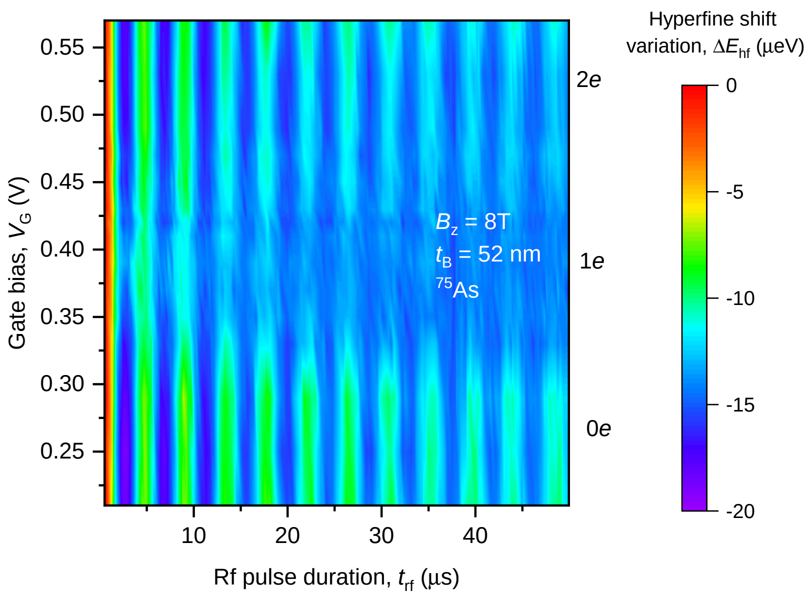

Supplementary Figure 4. **Bias dependant ^{75}As Rabi oscillations**. Rf induced variation of the hyperfine shift $\Delta E_{\rm hf}$ as a function of rf pulse duration $t_{\rm rf}$ measured at $B_z = 8$ T in the $t_{\rm B} = 52$ nm sample for a range of gate biases $V_{\rm G}$ across the single-electron $(1e)$ charging plateau centered at $V_{\rm G} \approx 0.4$ V. Each point is obtained by averaging over multiple measurements. Inhomogeneous Knight shifts and electron spin flips occurring during the rf pulse lead to dephasing, resulting in reduced oscillation amplitude in the single electron regime $(1e)$. In a doubly charged state $(2e, V_{\rm G} > 0.55$ V) Rabi oscillations are similar to oscillations in an empty dot $(0e, V_{\rm G} < 0.25$ V) confirming that the damping of the oscillation is due to the unpaired spin of a single electron.

FWHM of ≈ 53.8 kHz. The larger spectral broadening of ^{75}As is due to its larger quadrupolar moment and the stronger second order quadrupolar shifts arising from the lattice-constant-scale strain, induced by the random alloying of Ga and In atoms [3].

When a single electron is added to the dot we find for ^{69}Ga at $B_{\rm z} = 7.8$ T the dephasing time of $T_{2,\rm N}^{*,(1e)} \approx 4.3$ μs and a spectral profile with $\eta \approx 1.88$, and a FWHM of ≈ 126 kHz. For ^{75}As we find $T_{2,\rm N}^{*,(1e)} \approx 4.5$ μs, $\eta \approx 2.05$, and a FWHM of ≈ 116 kHz. The additional broadening due to electron charging characterises the magnitude and inhomogeneity of the electron-induced Knight shifts of the individual nuclei in the dot. The Knight shifts of ^{75}As are smaller due to its smaller gyromagnetic ratio and consequently smaller hyperfine constant compared to ^{69}Ga.

At a reduced magnetic field of $B_{\rm z} = 1.94$ T we find for ^{69}Ga the dephasing times of $T_{2,\rm N}^{*,(0e)} \approx 13.6$ μs and $T_{2,\rm N}^{*,(1e)} \approx 3.6$ μs. The spectral profile for an empty quantum dot is characterised by $\eta \approx 1.03$, and a FWHM of ≈ 23 kHz, which is twice larger than at high magnetic field, as expected for broadening related to second order quadrupolar effects [10]. For the nuclear spin

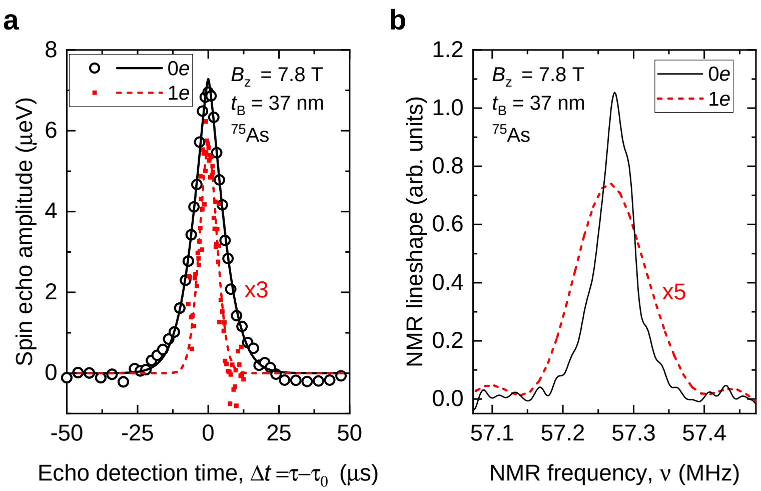

Supplementary Figure 5. **Free induction decay spectroscopy of ^{75}As nuclei in a single quantum dot. a,** Spin echo amplitude measured with rf pulse sequence shown in Fig. 1b of the main text and plotted as a function of the second delay τ for a constant first delay τ_0. Results are for the $t_{\mathrm{B}} = 37$ nm sample at $B_{\mathrm{z}} = 7.8$ T, and show the free induction decay in $0e$ (circles, $\tau_0 = 150$ μs) and $1e$ (squares, $\tau_0 = 7.5$ μs, data multiplied is by 3) charge states. Lines show compressed exponential fitting used to derive the nuclear spin dephasing times $T^*_{2,\mathrm{N}}$. **b,** Fourier transform of **a**, data for $1e$ is multiplied by 5.

spectral lineshape in presence of a single electron we find $\eta \approx 2.04$, and a FWHM of ≈ 150 kHz, consistent with the results obtained at high fields. Unlike at high fields, the average Knight shift $\langle \nu_{\mathrm{e}} \rangle$ is very small at $B_{\mathrm{z}} = 1.94$ T due to the small equilibrium electron spin polarization. For ^{75}As at low magnetic fields the quadrupolar induced spectral broadening becomes too large to conduct reliable pulsed NMR spectroscopy.

Supplementary Note 5. DYNAMICAL DECOUPLING OF THE NUCLEAR SPIN ENSEMBLE IN PRESENCE OF A SINGLE ELECTRON SPIN.

Dynamical decoupling is a technique used to suppress decoherence arising from unwanted interactions of a quantum system. A commonly used implementation relies on a series of short (bang-bang) control pulses [14, 15] that transform the effective Hamiltonian governing the interactions in the free evolution intervals following each pulse. The control sequence can be chosen to selectively remove some of the interactions or can be chosen to implement 'time-suspension' where all the interactions are suppressed. Here we discuss experimental results on dynamical decoupling of the nuclear spins in an individual quantum dot in presence of a single electron ($1e$). The methodology follows the previous work [12] conducted on neutral QDs ($0e$ state). Each NMR pulse

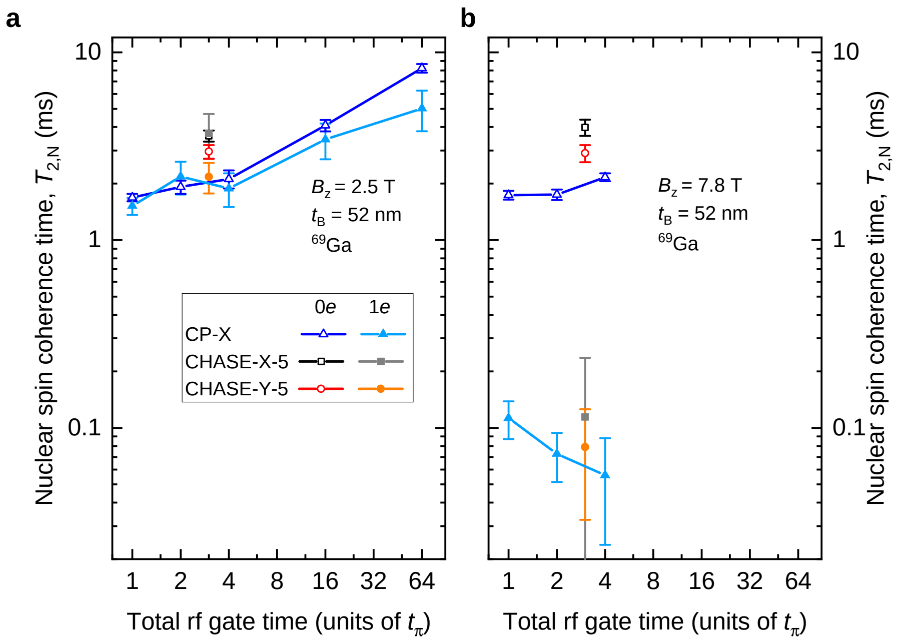

Supplementary Figure 6. **Coherence of ^{69}Ga nuclear spins under dynamical decoupling. a,** Measurements of $T_{2,\mathrm{N}}^{(0e)}$ (open symbols) and $T_{2,\mathrm{N}}^{(1e)}$ (solid symbols) for a QD in the $t_\mathrm{B} = 52$ nm sample at $B_\mathrm{z} = 2.5$ T. The coherence times are plotted as a function of the total rf sequence gate time in units of π-pulse duration $t_\pi \approx 1.4$ μs, excluding the initial and final pulses. **b,** Same as **a** but for $B_z = 7.8$ T. Error bars are 95% confidence intervals.

sequence starts with a $(\pi/2)_\mathrm{x}$ or $(\pi/2)_\mathrm{y}$ pulse creating transverse nuclear spin polarization along the x or y axis of the rotating frame, respectively (sequences labeled as -X and -Y, respectively). A dynamical decoupling sequence of rf pulses is then applied, followed by a final $\pi/2$ pulse of the same phase (same rotation axis) as the initial pulse to convert the preserved fraction of the nuclear spin coherence into an optically detectable longitudinal nuclear spin polarization. The decay of coherence as a function of the total free evolution time τ_evol between the initial and the final pulses is fitted with a stretched or compressed exponent to derive the coherence time $T_{2,\mathrm{N}}$.

We start by examining ^{69}Ga nuclei in the low field regime ($B_\mathrm{z} = 2.5$ T) in a $t_\mathrm{B} = 52$ nm sample. Under these conditions the electron spin flips, characterised by $T_{1,\mathrm{e}} \approx 10$ ms, are slow compared to dipolar-dominated nuclear spin coherence time $T_{2,\mathrm{N}}^{(0e)} \approx 1.69$ ms in an empty QD. For each decoupling sequence the resulting $T_{2,\mathrm{N}}$ is shown in Supplementary Fig. 6a as a function of the total duration of the control pulses, excluding the initial and final pulses, and measured in units of a π-pulse duration t_π. The Carr Purcell sequence (CP-X) consists of a train of periodic π pulses with the same phase as the initial and final pulses [12]. Spin echo is a particular case with just one π pulse. The CP sequence removes the dephasing induced by inhomogeneous broadening, but does

not affect the dipolar nuclear-nuclear interactions. For ideal (infinitely fast) control pulses and a static inhomogeneous broadening, $T_{2,\mathrm{N}}$ is expected to be constant for any number of π pulses. By contrast, in experiment on an empty QD ($0e$, open triangles) we find an increase in $T_{2,\mathrm{N}}$ with an increasing number of π pulses applied during the τ_{evol} interval. This increase in $T_{2,\mathrm{N}}$ is a pulsed spin locking effect [12, 16] resulting from coherent evolution of the interacting nuclear spins during the non-ideal (finite-duration) rf control pulses. In presence of an electron ($1e$, solid triangles) spin locking is observed as well, although the maximum $T_{2,\mathrm{N}}$ is smaller than in the $0e$ case, most likely limited by $T_{2,\mathrm{N}}$ approaching the $T_{1,\mathrm{e}}$, when it becomes probable that the nuclear spin dynamical decoupling echo is destroyed by an electron spin flip occurring during the free evolution interval τ_{evol}.

To implement the time-suspension we use CHASE-5 sequence [12], which suppresses both the ensemble inhomogeneous broadening and the nuclear-nuclear interactions. Unlike the spin locking, which preserves the coherence only along the preferential direction of the rotating frame, the CHASE sequence is designed to preserve a coherent state with an arbitrary phase. This is verified by measuring the decay of the echo initialised either along the x or the y axis of the rotating frame, with results shown in Supplementary Fig. 6a by the squares and circles, respectively. In agreement with previous studies [12], in an empty QD ($0e$) CHASE-5 leads to approximately a factor of 2 increase in coherence time, from $T_{2,\mathrm{N}}^{(0e)} \approx 1.69 \pm 0.085$ ms for spin echo to $T_{2,\mathrm{N}}^{(0e)} \approx 3.59 \pm 0.24$ ms and $T_{2,\mathrm{N}}^{(0e)} \approx 2.96 \pm 0.24$ ms for CHASE-X-5 and CHASE-Y-5 sequences, respectively. In presence of the electron spin ($1e$) CHASE-X-5 is equally effective in improving the spin echo coherence time $T_{2,\mathrm{N}}^{(1e)} \approx 1.50 \pm 0.15$ ms to $T_{2,\mathrm{N}}^{(1e)} \approx 3.7 \pm 1.0$ ms (solid square in Supplementary Fig. 6a). In the worst case scenario of a coherent state with polarisation along the y axis of the rotating frame (CHASE-Y-5, solid circle in Supplementary Fig. 6a) the observed $T_{2,\mathrm{N}}^{(1e)} \approx 2.2 \pm 0.4$ ms is still a significant improvement over the single-pulse spin echo. Longer decoupling sequences, such as CHASE-10 and CHASE-20 lead to accumulation of pulse control errors, and reduction of the echo amplitude which prevents reliable $T_{2,\mathrm{N}}^{(1e)}$ measurement. Nevertheless, the CHASE-5 results confirm that the central spin is not a fundamental obstacle for dynamical decoupling of the nuclear spin bath.

In the high field regime ($B_{\mathrm{z}} = 7.8$ T, Supplementary Fig. 6b) the addition of the electron spin to the quantum dot results in a pronounced reduction of the ^{69}Ga nuclear spin coherence. This is expected since the significantly shortened $T_{1,\mathrm{e}} \approx 90$ μs makes spectral diffusion mechanism dominant over the intrinsic dipolar-induced decoherence. The accuracy is not sufficient to evaluate the trends in $T_{2,\mathrm{N}}^{(1e)}$ as a function of the total rf gate time, but it can be seen that neither CP

nor CHASE sequences provide any reliable improvement in $T_{2,\mathrm{N}}^{(1e)}$, suggesting that both the pulsed spin locking and time-suspension are disrupted. The mechanism is similar to the one that governs the spin echo decoherence under spectral diffusion in the limit of slow fluctuations (see details in Supplementary Note 2). Owing to the strong Knight shift inhomogeneity, characterised by short dephasing time $T_{2,\mathrm{N}}^{*,(1e)}$, the probability that a single electron flip destroys the nuclear spin echo $\approx 1 - T_{2,\mathrm{N}}^{*,(1e)}/T_{2,\mathrm{N}}^{(0e)}$ is close to unity. This is also the case for CP and CHASE sequences: just as for spin echo, these dynamical decoupling sequences rely on rf pulses periodically transforming the instantaneous interaction Hamiltonian to converge the average Hamiltonian to zero. A single electron flip is sufficient to disrupt the decoupling by breaking the balance of phases accumulated by spins in the free evolution intervals of the sequence. As a result, nuclear spin coherence is essentially limited by the electron spin lifetime $T_{2,\mathrm{N}}^{(1e)} \lesssim 1.38T_{1,\mathrm{e}}$. Under these conditions, an improvement of $T_{2,\mathrm{N}}^{(1e)}$ with CHASE can be expected only if a large number of decoupling pulses is applied with intervals much shorter than the timescale $T_{1,\mathrm{e}}$ of the electron spin flips. This regime is currently not achievable in our experiments on ^{69}Ga, and would require improvement of the echo amplitude through optimisation of the rf circuits to attain larger rf magnetic field while reducing the parasitic rf electric field.

We also examine dynamical decoupling of ^{75}As nuclei in the high field regime ($B_\mathrm{z} = 7.8$ T). The results shown in Supplementary Fig. 7 are consistent with the high field results for ^{69}Ga (Supplementary Fig. 6b), showing that dynamical decoupling provides no reliable improvement when nuclear spin decoherence is dominated by random electron spin flips rather than by the nuclear spin-spin interactions. Due to the 100% natural abundance, ^{75}As gives a larger NMR signal making it possible to measure $T_{2,\mathrm{N}}^{(1e)}$ under longer decoupling sequences than in ^{69}Ga. From these measurements shown in Supplementary Fig. 7 we observe a non-monotonic dependence of $T_{2,\mathrm{N}}^{(1e)}$ on the number of CP-X cycles (solid triangles). The coherence time first decreases to $T_{2,\mathrm{N}}^{(1e)} \approx 65$ μs under a sequence with 4 and 8 π-pulses. Such decrease can be ascribed to the "heating" of the spins by frequent control pulses, which reenables the dipolar flip-flops, otherwise frozen by inhomogeneous quadrupolar shifts [12, 17]. The coherence is then seen to increase to $T_{2,\mathrm{N}}^{(1e)} \approx 110$ μs under 16 π-pulses. This increase would be consistent with dynamical decoupling overcoming the random electron spin flips by periodically inverting the nuclear spins faster than $T_{1,\mathrm{e}}$. However, validation of these trends in $T_{2,\mathrm{N}}^{(1e)}$ would require further studies with improved measurement accuracy.

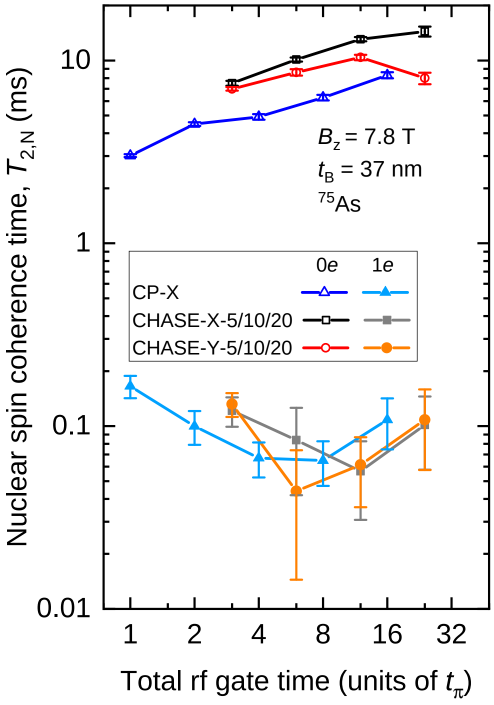

Supplementary Figure 7. **Coherence of ^{75}As nuclear spins under dynamical decoupling. a,** Measurements of $T_{2,\mathrm{N}}^{(0e)}$ (open symbols) and $T_{2,\mathrm{N}}^{(1e)}$ (solid symbols) on a QD in the $t_\mathrm{B} = 37$ nm sample at $B_\mathrm{z} = 7.8$ T. The coherence times are plotted as a function of the total rf sequence gate time in units of π-pulse duration $t_\pi \approx 2.1$ μs with initial and final pulses excluded. The points at $3t_\pi$ correspond to one cycle of CHASE-5 sequence, the points at $6t_\pi$ correspond to one cycle of CHASE-10 and the points at $\geq 12t_\pi$ are for integer numbers of CHASE-20 cycles. Error bars are 95% confidence intervals.

Supplementary Note 6. TABULATED DATA

Here we present tabulated data for the nuclear spin echo coherence times $T_{2,\mathrm{N}}$ measured as a function of magnetic field B_z under $0e$ and $1e$ quantum dot charging states in samples with different tunnel barriers t_B. The same results are shown by the symbols in Fig. 2a of the main text. Error estimates are 95% confidence intervals. Results for ^{69}Ga in a $t_\mathrm{B} = 52$ nm sample:

B_z (T)	$T_{2,\mathrm{N}}^{(0e)}$ (μs)	$T_{2,\mathrm{N}}^{(1e)}$ (μs)
1.44196	1700.72 ± 107.563	1419.69 ± 440.492
2.50368	1685.17 ± 78.661	1520.81 ± 152.137
4.60078	1597.59 ± 103.007	410.265 ± 109.825
7.86045	1737.51 ± 88.2604	112.902 ± 26.1098

(9)

Results for ^{69}Ga in a $t_{\rm B} = 37$ nm sample:

B_z (T)	$T_{2,\rm N}^{(0e)}$ (μs)	$T_{2,\rm N}^{(1e)}$ (μs)
0.2869	1744.9 ± 119.158	865.063 ± 169.133
0.5924	1815.77 ± 113.388	717.886 ± 81.3125
1.94212	1846.53 ± 91.5654	780.489 ± 136.701
3.3608	2075.52 ± 124.196	875.626 ± 188.566
4.56608	1986.71 ± 83.448	585.836 ± 135.94
5.56498	1934.72 ± 86.5221	376.924 ± 91.9341
6.44144	2001.8 ± 90.4204	201.962 ± 42.5813
7.79952	1974.6 ± 74.5993	135.675 ± 36.2282

(10)

Results for ^{75}As in a $t_{\rm B} = 52$ nm sample:

B_z (T)	$T_{2,\rm N}^{(0e)}$ (μs)	$T_{2,\rm N}^{(1e)}$ (μs)
7.81457	2937.5 ± 104.373	134.56 ± 18.4758

(11)

Results for ^{75}As in a $t_{\rm B} = 37$ nm sample:

B_z (T)	$T_{2,\rm N}^{(0e)}$ (μs)	$T_{2,\rm N}^{(1e)}$ (μs)
7.81661	3016.07 ± 103.639	165.165 ± 17.8594

(12)

Results for ^{71}Ga in a $t_{\rm B} = 37$ nm sample:

B_z (T)	$T_{2,\rm N}^{(0e)}$ (μs)	$T_{2,\rm N}^{(1e)}$ (μs)
7.7628	1348.35 ± 72.4856	141.483 ± 21.1552

(13)

SUPPLEMENTARY REFERENCES

[1] Slichter, C. P. *Principles of Magnetic Resonance* (Springer, 1990).

[2] Bulutay, C. Quadrupolar spectra of nuclear spins in strained $\mathrm{In}_x\mathrm{Ga}_{1-x}\mathrm{As}$ quantum dots. *Phys. Rev. B* **85**, 115313 (2012).

[3] Chekhovich, E. A. *et al.* Structural analysis of strained quantum dots using nuclear magnetic resonance. *Nat. Nanotechnol.* **7**, 646–650 (2012).

[4] Gueron, M. Density of the conduction electrons at the nuclei in indium antimonide. *Phys. Rev.* **135**, A200–A205 (1964).

[5] Gotschy, B., Denninger, G., Obloh, H., Wilkening, W. & Schnieder, J. Overhauser shift and dynamic nuclear polarization in InP. *Solid State Commun.* **71**, 629–632 (1989).

[6] Chekhovich, E. A. *et al.* Measurement of the spin temperature of optically cooled nuclei and GaAs hyperfine constants in GaAs/AlGaAs quantum dots. *Nat. Mater.* **16**, 982 (2017).

[7] Klauser, D., Coish, W. A. & Loss, D. Nuclear spin dynamics and Zeno effect in quantum dots and defect centers. *Phys. Rev. B* **78**, 205301 (2008).

[8] Reilly, D. J. *et al.* Exchange control of nuclear spin diffusion in a double quantum dot. *Phys. Rev. Lett.* **104**, 236802 (2010).

[9] Gillard, G. *et al.* Fundamental limits of electron and nuclear spin qubit lifetimes in an isolated self-assembled quantum dot. *npj Quantum Inf.* **7**, 43 (2021).

[10] Chekhovich, E. A., Hopkinson, M., Skolnick, M. S. & Tartakovskii, A. I. Suppression of nuclear spin bath fluctuations in self-assembled quantum dots induced by inhomogeneous strain. *Nat. Commun.* **6**, 6348 (2015).

[11] Wüst, G. *et al.* Role of the electron spin in determining the coherence of the nuclear spins in a quantum dot. *Nat. Nanotechnol.* **11**, 885–889 (2016).

[12] Waeber, A. M. *et al.* Pulse control protocols for preserving coherence in dipolar-coupled nuclear spin baths. *Nat. Commun.* **10**, 3157 (2019).

[13] Haase, J. & Conradi, M. S. Sensitivity enhancement for NMR of the central transition of quadrupolar nuclei. *Chem. Phys. Lett.* **209**, 287–291 (1993).

[14] Waugh, J. S., Huber, L. M. & Haeberlen, U. Approach to High-Resolution NMR in Solids. *Phys. Rev. Lett.* **20**, 180–182 (1968).

[15] Viola, L., Knill, E. & Lloyd, S. Dynamical decoupling of open quantum systems. *Phys. Rev. Lett.* **82**, 2417–2421 (1999).

[16] Li, D. *et al.* Intrinsic origin of spin echoes in dipolar solids generated by strong π pulses. *Phys. Rev. B* **77**, 214306 (2008).

[17] Waeber, A. M. *et al.* Few-second-long correlation times in a quantum dot nuclear spin bath probed by frequency-comb nuclear magnetic resonance spectroscopy. *Nat. Phys.* **12**, 688–693 (2016).
